# Supplementary material for: Multi‐dataset identification of innovative feature genes and molecular mechanisms in keratoconus
Source: J Cell Mol Med. 2024 Sep 19;28(18):e70079. doi: 10.1111/jcmm.70079 (PMC11412914; doi:10.1111/jcmm.70079)
Supplement: Supplementary file 2 — Material S2. [file JCMM-28-e70079-s002.zip › 7.GSEA analysis/2.ATOH7/KEGG_ABC_TRANSPORTERS.html]

Details for gene set KEGG\_ABC\_TRANSPORTERS[GSEA]

|  || Dataset | ATOH7.group.cls#h\_versus\_l |
| Phenotype | group.cls#h\_versus\_l |
| Upregulated in class | l |
| GeneSet | KEGG\_ABC\_TRANSPORTERS |
| Enrichment Score (ES) | -0.19083203 |
| Normalized Enrichment Score (NES) | -0.5697592 |
| Nominal p-value | 1.0 |
| FDR q-value | 1.0 |
| FWER p-Value | 1.0 |
Table: GSEA Results Summary

  

Fig 1: Enrichment plot: KEGG\_ABC\_TRANSPORTERS      
 Profile of the Running ES Score & Positions of GeneSet Members on the Rank Ordered List

  

| SYMBOL | TITLE | RANK IN GENE LIST | RANK METRIC SCORE | RUNNING ES | CORE ENRICHMENT || 1 | ABCD2 | na | 726 | 0.494 | 0.0705 | No |
| 2 | ABCG2 | na | 2334 | 0.321 | 0.0835 | No |
| 3 | ABCC1 | na | 3133 | 0.274 | 0.1112 | No |
| 4 | ABCG4 | na | 3871 | 0.241 | 0.1346 | No |
| 5 | CFTR | na | 4476 | 0.219 | 0.1577 | No |
| 6 | ABCG8 | na | 5575 | 0.183 | 0.1600 | No |
| 7 | ABCA13 | na | 6615 | 0.154 | 0.1585 | No |
| 8 | ABCC3 | na | 7455 | 0.132 | 0.1587 | No |
| 9 | ABCB11 | na | 8638 | 0.106 | 0.1442 | No |
| 10 | ABCA2 | na | 9407 | 0.092 | 0.1391 | No |
| 11 | ABCC4 | na | 11571 | 0.058 | 0.0874 | No |
| 12 | ABCC11 | na | 11881 | 0.054 | 0.0885 | No |
| 13 | ABCA10 | na | 11921 | 0.054 | 0.0973 | No |
| 14 | ABCG5 | na | 13087 | 0.039 | 0.0710 | No |
| 15 | ABCB6 | na | 13414 | 0.036 | 0.0683 | No |
| 16 | TAP1 | na | 14381 | 0.027 | 0.0454 | No |
| 17 | TAP2 | na | 14637 | 0.024 | 0.0426 | No |
| 18 | ABCC6 | na | 15731 | 0.015 | 0.0138 | No |
| 19 | ABCB9 | na | 15854 | 0.014 | 0.0129 | No |
| 20 | ABCA7 | na | 17065 | 0.004 | -0.0213 | No |
| 21 | ABCB10 | na | 17409 | 0.001 | -0.0310 | No |
| 22 | ABCG1 | na | 17554 | -0.000 | -0.0351 | No |
| 23 | ABCB7 | na | 18995 | -0.013 | -0.0742 | No |
| 24 | ABCA12 | na | 19294 | -0.015 | -0.0800 | No |
| 25 | ABCC5 | na | 20994 | -0.032 | -0.1231 | No |
| 26 | ABCC12 | na | 21018 | -0.032 | -0.1179 | No |
| 27 | ABCD1 | na | 21355 | -0.036 | -0.1210 | No |
| 28 | ABCA1 | na | 22141 | -0.045 | -0.1354 | No |
| 29 | ABCA5 | na | 22707 | -0.051 | -0.1422 | No |
| 30 | ABCD3 | na | 23572 | -0.063 | -0.1554 | No |
| 31 | ABCC10 | na | 23615 | -0.064 | -0.1448 | No |
| 32 | ABCB8 | na | 24441 | -0.076 | -0.1545 | No |
| 33 | ABCB1 | na | 24737 | -0.081 | -0.1479 | No |
| 34 | ABCA6 | na | 26226 | -0.109 | -0.1706 | Yes |
| 35 | ABCD4 | na | 26495 | -0.115 | -0.1571 | Yes |
| 36 | ABCA3 | na | 27028 | -0.126 | -0.1491 | Yes |
| 37 | ABCC9 | na | 27470 | -0.137 | -0.1364 | Yes |
| 38 | ABCA8 | na | 28583 | -0.167 | -0.1376 | Yes |
| 39 | ABCC2 | na | 29054 | -0.181 | -0.1177 | Yes |
| 40 | ABCA9 | na | 29492 | -0.194 | -0.0943 | Yes |
| 41 | ABCB5 | na | 29673 | -0.200 | -0.0626 | Yes |
| 42 | ABCC8 | na | 31416 | -0.265 | -0.0638 | Yes |
| 43 | ABCA4 | na | 31814 | -0.284 | -0.0227 | Yes |
| 44 | ABCB4 | na | 34312 | -0.576 | 0.0120 | Yes |
Table: GSEA details [plain text format]

  

Fig 2: KEGG\_ABC\_TRANSPORTERS      
 Blue-Pink O' Gram in the Space of the Analyzed GeneSet

  

Fig 3: KEGG\_ABC\_TRANSPORTERS: Random ES distribution      
 Gene set null distribution of ES for **KEGG\_ABC\_TRANSPORTERS**
